# Transitioning to digital first line intervention – validation of a brief online screener for early identification of a suspected eating disorder: study protocol

**DOI:** 10.1186/s40337-020-00339-8

**Published:** 2020-11-12

**Authors:** Emma Bryant, Jane Miskovic-Wheatley, Stephen Touyz, Ross D. Crosby, Eyza Koreshe, Li Cao, Sarah Maguire

**Affiliations:** 1 School of Medical Sciences, Faculty of Medicine and Health, The University of Sydney, Sydney, Australia; 2 InsideOut Institute for Eating Disorders, The Boden Collaboration for Obesity, Nutrition, Exercise and Eating Disorders, The University of Sydney, Sydney, Australia; 3 Sanford Center for Biobehavioral Research, Sanford Research, Fargo, ND USA

**Keywords:** Eating disorders, Early intervention, COVID-19, Psychometrics, Telehealth, Digital, Screening questionnaire

## Abstract

**Background:**

Only one in four people with eating disorders seeks treatment, and of those who do seek treatment, 20% go on to experience a chronic course. Early intervention has been associated with better prognosis, with those seeking specialised intervention in the early stages of their illness more than twice as likely to achieve remission. Current screening measures typically require expert administration and are rarely validated across a spectrum of DSM-5 eating disorder presentations or for online use. In light of COVID-19 and increasing reliance on telehealth technologies in the intervention and delivery of mental health services, online assessments suitable for self-referral are likely to be the first step to seeking care. InsideOut Institute has developed a 6-item online screening tool for the purposes of identifying eating disorder risk and symptomatology, aimed specifically at increasing help-seeking behaviour in subsyndromal and early presentations.

**Methods:**

This study investigates the reliability and validity of the InsideOut Institute Screener (IOI-S), using a cross-sectional survey research design. Participants aged 14 and over will complete an extensive baseline survey battery for evaluation. 50% of participants will be randomly selected for one follow-up re-test of the IOI-S only, 2 weeks post initial testing. The IOI-S will be analysed for statistical reliability on two parameters: internal consistency and test re-test reliability, and for statistical validity on four parameters: concurrent validity, sensitivity and specificity, convergent and discriminant validity.

**Discussion:**

The rapid and ongoing shift to digital intervention has highlighted gaps and opportunities in our pathways to care. Adequate screening for eating disorders is a major gap. This study aims to validate an online screening tool for use in telehealth early intervention, designed for users seeking information for a suspected eating disorder. The screener meets those at risk ‘where they are’ (i.e. online) and may improve timely referrals to relevant services. This is of particular salience as face-to-face healthcare and traditional frontline interventions are disrupted, and we are challenged to re-design our practices to deliver diagnostic and treatment services in highly adaptive digital contexts.

## Plain English summary

Early intervention greatly improves treatment outcome, however currently only 1 in 4 people with an eating disorder seeks treatment. Current screening measures are typically designed to identify traditional cases of Anorexia and Bulimia Nervosa rather than address a spectrum of eating disorder behaviour and are rarely validated for online use. Adequate screening designed to encourage help-seeking behaviour is needed. Given 84% of Australians now seek healthcare information online before going anywhere else, and with a rapid COVID-19 driven shift to digital healthcare and intervention, novel online screening tools designed to identify broad eating disorder risk and symptomatology may become the first step to seeking care. This study aims to validate a 6-item online screening tool developed by the InsideOut Institute for Eating Disorders at the University of Sydney in a diverse population of people aged 14 and over. It is designed to ‘start a conversation’ so that it may be used to encourage help-seeking behaviour and improve timely referrals to relevant services (digital or otherwise).

## Background

Eating Disorders (ED), marked by persistent disturbed eating behaviour and negative psychological affect, are complex psychiatric conditions associated with life-threatening physical complications [1] and high concurrent mood disorders [2, 3]. Anorexia (AN) and Bulimia Nervosa (BN) alone cause 1.9 million disability-adjusted life years globally [4] and cost the Australian economy $69.7 billion per year [5]. Despite this, only one in four people with EDs seek treatment [6], and of those who seek treatment, 20 % will become chronic [7]. Elevated mortality rates and relatively poor treatment outcomes may in part be attributable to a paucity in help-seeking and the frequent delay between illness onset and intervention – a factor known to heavily influence prognosis [8, 9].

Early intervention is paramount to improved treatment outcome [10, 11], however is frequently impeded by mental health illiteracy, self-stigma and shame [12]. Eating disorder stereotypes exacerbate ambivalence towards treatment seeking [13] and contribute to the ego-syntonic nature of the illness, where people can be stuck between the pre-contemplation (denial) and contemplation (awareness with resistance) stages as described by Prochaska’s trans-theoretical model of change [14]. This ambivalence may be compounded by cultural normalisation of dietary and exercise excess, contributing to confusion around experiences of pathology and distress [15].

Screening tools are regularly used to triage medical and psychological risk in healthcare settings and have been shown to be effective in early identification, often triggering referral and further assessment where results suggest a condition may be present [16]. They may have superior utility over long form diagnostic tools in the crucial pre-contemplative stage of an eating disorder, mitigating the challenges clients frequently face in being properly identified by time-poor primary healthcare physicians, many of whom lack the expertise or feel ill-equipped to deliver complex eating disorder assessments [17, 18] such as the gold standard Eating Disorder Examination Questionnaire [19, 20].

A number of screener questionnaires have been validated for use in eating disorders. These include the widely used 5-item Sick, Control, One Stone, Fat, Food (SCOFF) questionnaire (Morgan, Reid & Lacey, [21]) and the 5-item Eating Disorder Screen for Primary Care (ESP) (Cotton, Ball & Robinson, [22]). The SCOFF is brief and has demonstrated good internal consistency and concurrent validity with the EDE-Q, however, shows limited evidence identifying disordered behaviour beyond traditional presentations of AN and BN [23]. Additionally, language may assume acceptance of an eating disorder diagnosis, thus may not be useful in the prodromal phase of the illness. The SCOFF has attracted criticism for its use of confronting behaviour-based questions (e.g. ‘Do you make yourself sick?’, ‘Have you lost more than one stone?’) [23]. These types of questions have been found to be prone to denial or distortion on face-to-face assessment [24]. Moreover, the screener has limited specificity—the authors agreed maximising sensitivity was paramount given the morbidity and mortality associated with eating disorders and the consequences of a false negative result, thus reducing the cut-off from three positive answers to two [21]_._ Importantly, as with most existing screeners, the SCOFF is validated for in-person use only, and thus assumes a person is already in the care of a healthcare professional at the time of taking the test.

The Eating Disorder Screen for Primary Care is more sensitive but less specific than SCOFF at detecting eating disorders [25]. It employs more sympathetic language and includes questions about personal and family history of eating disorder diagnoses, however the latter has been found to have no predictive strength and is dropped from analyses in many cases [25]. The ESP has not been widely circulated or validated.

Several short forms of the EDE-Q, including the EDE-QS (Gideon et al., [26]) (12 items), the EDE-Q7 (Grilo, Henderon, Bell & Crosby, [27]) and the EDE-Q8 (Kliem et al., [28]) have been conceived and psychometrically validated across populations, demonstrating good sensitivity and internal consistency and adequately correlating with the original EDE-Q. However, all utilise the original items, derived from previous iterations of the DSM and prior to the inclusion of diagnostic categories Binge Eating Disorder (BED), Avoidant Restrictive Food Intake Disorder (ARFID), Other Specified Feeding or Eating Disorder (OSFED) and Atypical AN, and again, are not specifically designed for online use.

To our knowledge, there have been few intermediary measures and novel screening tools developed since the 2013 revision of the DSM (5) despite changes to existing diagnostic categories and the inclusion of a BED diagnosis. Further, existing diagnostic and screening measures are typically validated to discriminate between healthy and ill, rather than using a continuum to identify those showing early symptomatology/risk [29]. One recent exception is the Stanford-Washington University Eating Disorder screen (SWED) (Graham et al., [30]) which assigns a risk threshold and has been validated for online use [30, 31]. However, this questionnaire is 17 items long and has been primarily validated in college age females. Given the prognostic implications of early intervention, there is evidenced need to expand on current validated screeners for diverse populations, and to do so in a way that reflects modern-day healthcare and the move to online health information-seeking [32].

Healthcare is experiencing a paradigmatic shift from medical paternalism to autonomous patient-centred care [33] with people increasingly turning to internet technologies for support and advice [34]. Eighty-four percent of Australians log on to the internet to search healthcare information before going anywhere else [35]. The most at-risk population for eating disorders, young people, are significantly more likely to seek initial information online than to visit their GP [36]. The occasion of the COVID-19 global pandemic has driven a sudden shift to online healthcare delivery [37], alongside increased rates of mental health complaints and worsening anxiety and depression [38, 39]. Those suffering from eating disorders are particularly vulnerable during times of food insecurity, with lack of access to “safe” foods combined with erratic food distribution causing a ‘feast or famine’ pattern defined by hoarding, restriction, bingeing and compensation [40, 41]. There is evident emergent need, then, for novel online risk-assessments and screening options suitable for self-referral, where the burden of mental health is increasing while face-to-face access to physicians is curtailed by social distancing measures [42, 43]. New screening tools should address the semantic and methodological limitations of present standardised measures and be validated for online use across the spectrum of eating disorders as defined by the DSM-5 (including presentations of BED, ARFID and OSFED). They may be more acceptable and less susceptible to denial and distortion by employing non-confrontational language [24]. To this end, InsideOut Institute for Eating Disorders (IOI) within the Boden Collaboration for Obesity, Nutrition, Exercise and Eating Disorders, The University of Sydney, conceptualised a short, 6-item online screening tool, the InsideOut Institute Screener (IOI-S, [44]) to assess for eating disorder symptomatology/risk using non-intrusive, sensitive language designed to ‘start a conversation.’

This study aims to psychometrically validate the IOI online screener so that it may be used reliably as an adjunct or alternative to existing screening measures and/or for referral purposes, encouraging online users who may otherwise have delayed seeking professional help to do so with self-governance and autonomy.

## Methods/design

### Participants

Approximately 250 participants will be recruited into the study. They will be aged 14 and over, of all genders, and divided into two groups – clinical and non-clinical. Clinical status will be determined post-testing using EDE-Q cut-off scores. We aim to recruit across all DSM 5 diagnostic categories for eating disorders.

Participants will be recruited in two ways:

1. *Amazon’s Mechanical Turk (MTurk):* ‘MTurk’ is a global internet crowdsourcing service used frequently in academic research to recruit a broad base of willing research participants who are compensated for completing surveys, questionnaires and tasks as assigned by the researcher [45]. MTurk does not permit people under 18 to use its service: thus, it will be used to recruit adults aged 18+ only, both clinical and non-clinical. Participants will be able to sign up from anywhere in the world, deeming it an international cohort. They will be reimbursed according to MTurk recommendations.
2. *Community:* Participants will be recruited via online advertisements on health websites and social media channels/paper advertisements. Advertisements will be aimed at individuals who self-report an eating disorder diagnosis, though those without an eating disorder are also welcome to participate, and all will go into the draw to win a Westfield gift card. We are specifically looking to recruit adolescents via this method, due to MTurk’s restriction on underage participants, however adults will also be targeted.

The use of two methods of recruitment aims to increase generalisability across age, ethnicity, gender and Body Mass Index (all of which will be reported) – this is significant due to the broad-reaching nature of the internet and the importance of validating the questionnaire across populations.

### Inclusion/exclusion criteria

At this stage of validation, the screener will be conducted in the English language only, thus proficiency in reading the English language is required. Participants under the age of 14 are excluded from the study – separate validation studies are indicated for younger age groups due to differences in comparison measures, linguistic ability and potential impact on item comprehension. Also excluded are those with no reliable access to the internet. Otherwise, no further demographic or exclusion criteria will be set. Participants with a current diagnosis of an eating disorder as well as those with no current or former diagnosis are equally welcome to participate: these two groups (clinical and non-clinical) will be defined and compared post testing using EDE-Q cut-off scores.

### Overall study design

This study will use a cross-sectional survey research design with follow up on a sub-sample to examine the 6-item IOI-S for statistical reliability and validity. Participants will be given access to a baseline survey package delivered on the secure web survey platform REDCap. Approximately half the participants will be randomly selected to participate in a follow-up re-test involving completion of the IOI-S alone a second time 2 weeks post initial testing, to account for potential drop-out/uptake refusal. 30% is needed for sufficient test-retest reliability.

### Measures

Four questionnaires make up the baseline survey package: The EDE-Q, the SCOFF Questionnaire, the IOI-S and either the Marlowe-Crowne Social Desirability Scale (MC-SDS) (adults) or Children’s Social Desirability Short Scale (CSD-S) (adolescents), described in full below. The four existing surveys against which the IOI-S will be measured were chosen as the relevant diagnostic tools most frequently used in clinical settings and research trials. These will be presented in randomised order to the participants.

### Baseline package

#### InsideOut institute screener (IOI-S) (InsideOut Institute for Eating Disorders, [44])

The six items of the new scale are displayed in Table 1. Item development followed review of the scientific literature for existing instruments screening and assessing eating disorder symptomatology, lived experience and clinical and research expert consultation. Instruments reviewed included the EDE-Q, the Eating Disorders Inventory (Garner et al., [46]), the SCOFF questionnaire, the ESP, and the Eating Attitudes Test (EAT; Garner et al., [47]), from which researchers developed an initial pool of 10 questions covering six facets of eating pathology: an individual’s relationship with food, body, the extent to which body weight and shape determines self-worth, loss of control over eating, binge eating and compensatory behaviour. This was further narrowed by an expert consultation team to six relevant items to evaluate eating pathology and eating disorder risk in a non-clinical population. The IOI-S is rated on a 5-point Likert scale, where 1 is “never” and 5 is “all the time”; except for Question 1, where 1 is “worry and stress free” and 5 is “full of worry and stress”. Responses are summed to yield a score between 6 and 30 points total, where 6 points is the lowest degree of risk and 30 points the highest degree of risk. Those deemed to be of moderate to high risk are directed to InsideOut’s database of trained professionals with expertise in eating disorders.

**Table 1:** 6-item insideout institute screener

Item	1	2	3	4	5
1. How is your relationship with food?	Worry and stress-free	A bit problematic	Moderately problematic	Very problematic	Full of worry and stress
2. Does your weight, body or shape make you feel bad about yourself?	Never	A little bit	Sometimes	Quite a bit	All the time
3. Do you feel like food, weight or your body shape dominates your life?	Never	A little bit	Sometimes	Quite a bit	All the time
4. Do you feel anxious or distressed when you are not in control of your food?	Never	A little bit	Sometimes	Quite a bit	All the time
5. Do you ever feel like you will not be able to stop eating or have lost control around food?	Never	A little bit	Sometimes	Quite a bit	All the time
6. When you think you have eaten too much, do you do anything to make up for it?	Never	A little bit	Sometimes	Quite a bit	All the time

#### Eating disorder examination questionnaire (EDE-Q) (Fairburn & Beglin, [20])

The EDE-Q is a 28-item self-report version of the EDE structured clinical interview which is regarded as the ‘gold-standard’ diagnostic tool in eating disorders and has been validated in multiple trials [48]. Traditionally paper-based, it has been psychometrically validated for online delivery [49]. It includes additional measures of weight, height, and missed menstrual periods, the latter of which will be excluded from our study due to the DSM-5 removal of and consequent diagnostic irrelevance of missed menstruation in ED. It is comprised of a global score and four subscales: Restraint, Eating Concern, Weight Concern, and Shape Concern, and employs a 7-point forced-choice severity rating where 0 points is the lowest severity and 6 points is the highest severity.

#### SCOFF (sick, control, one stone, fat, food) questionnaire (Morgan et al., [21])

The SCOFF questionnaire is a short 5-item forced choice (true/false) screening tool designed to assess eating disorder symptomatology. A threshold of > 2 positive answers indicates a ‘likely case’ of AN or BN. It has shown good internal consistency and concurrent validity with the EDE-Q [50]. The SCOFF is included due to the frequency with which it has been employed in clinical and research settings.

#### Eating Disorder Examination Questionnaire 8 (EDE-Q8) (Kliem et al., [28]

The EDE-Q8 is an 8-item short form of the longer EDE-Q, designed as an abbreviated outcome measure that retains the original factor structure. It is highly correlated with the EDE-Q and demonstrates strong internal consistency, concurrent and convergent validity. Items are drawn directly from and are identical to respective items in the EDE-Q and will be extracted for independent analysis in this instance.

#### Marlowe-Crowne Social Desirability Scale (MC-SDS) (Crowne & Marlowe, [51])

The MC-SDS is a well-validated 33-item self-report questionnaire measuring social desirability in adults by evaluating concern with social approval. Items are rated true/false and balanced for positive and negative wording. It is frequently used as a measure of discriminant validity in instrument design [52] and has shown good divergent validity with measures of psychopathology (anxiety, depression) related to eating disorders [53]. The MC-SDS is not validated for use in children: thus, an adapted Children’s Social Desirability Scale (CSD-S) will apply for those participants aged under 18.

#### Children’s Social Desirability Short Scale (CSD-S) (Baxter et al, [54])

The CSD-S is an abbreviated 14-item version of the gold-standard 48-item Children’s Social Desirability scale developed by Crandall, Crandall and Katovsky in [55]. The original scale was modelled on the MC-SDS for adults and is validated for use in a child/adolescent population [54]. It will be assigned to our 14 to 17-year-old participants. The scale uses binary response (yes/no) and is regularly used for methodological validity to detect confounding from social desirability bias. The CSD-S has demonstrated adequate internal consistency and test-retest reliability and good external validity [56].

### Follow-up package

#### InsideOut institute screener (IOI-S) (InsideOut Institute for Eating Disorders, [44])

Follow-up involves completion of the 6-item IOI-S only.

### Sample size calculation

Estimated sample size is based on 2.5–3 times the minimum sample size required for power as the outcome measure (statistical validity) is incrementally increased with participant numbers. Therefore, we anticipate a total sample size of approximately 250 participants.

Approximately 30% of participants in each of the clinical and non-clinical groups (defined post T1 on EDE-Q cut-off score) will need to engage in the second task for sufficient validity. Estimated minimum sample size is based on the following analysis: in each group 10 participants are required for a power of 0.80 to detect an intraclass correlation coefficient of 0.70; 80 participants are required for sufficient power to detect a Spearman’s correlation of 0.40 or higher; 80 participants are required for good power to detect an Area Under Curve of 0.70, and at least 50 participants for a correlation coefficient of r +/− 0.70 for convergent and discriminant validity. All power analyses were conducted using PASS version 15 software [57].

### Data analyses

#### Reliability

Reliability is an important feature of survey efficacy indicating the overall replicability and internal stability of a measure [58]. Measure reliability of the IOI-S will be examined on two parameters: internal consistency and test-retest reliability. The internal consistency, that is, the degree to which the six items on the scale measure the same underlying dimension, will be calculated using a standardized Cronbach’s alpha score, with a minimum acceptable threshold of > .80 [59]. An exploratory factor analysis will be performed on the IOI-S items using principal axis factoring and Promax oblique rotation to examine factor loading, with an item-total correlation threshold of >.40 [60, 61]. Correlations between successive iterations of the test (test-retest reliability) will be reviewed using a 2-way mixed effects model Intraclass correlation coefficient with absolute agreement and a minimum acceptable threshold of > 0.70 [62].

#### Criterion validity

The degree to which the results on the IOI-S are able to predict an outcome on established measures of eating disorder symptomatology (concurrent validity) [63] will be assessed as concordance between the IOI-S, the EDE-Q, and the SCOFF using a Spearman’s product moment correlation. Following, an Area Under Curve (AUC) analysis of Receiver Operating Characteristic (ROC) curve will be performed as analysis of IOI-S sensitivity and specificity threshold where a high sensitivity indicates accurate identification of true positive cases and high specificity indicates accurate identification of true negative cases [64]. Area Under Curve dictates the probability of identifying true positives or negatives by chance, where a value of 0.50 would suggest the measure is no more effective than guessing [65]. AUC threshold will be set at > 0.70 with a 95% confidence interval.

#### Construct validity

Convergent validity, or the degree to which the IOI-S correlates with eating disorder constructs and variables on both the EDE-Q and the SCOFF [66] will be measured with a correlation coefficient threshold set at *r >* 0.70. A correlation coefficient will also be determined for discriminant validity between the IOI-S and MC-SDS/CSD-S. A correlation coefficient of +/− 0.70 indicates a strong linear relationship between two variables [67].

#### Missing and outlier values

REDCap online survey platform can be configured to eliminate missing values, otherwise submission of the survey battery is not possible. If data is not normally distributed, Spearman’s Rank Order will be used instead of Pearson’s correlation coefficient.

## Discussion

The internet is changing the way we seek and deliver healthcare, and current circumstances brought on by the COVID-19 global pandemic have intensified the need for validated online interventions and services, particularly where appropriate intervention at first point of contact is shown to be of fundamental gain to outcome. The primary outcome of this study is to establish if the Inside Out Institute Screener is a statistically reliable, valid and accurate online measure of eating disorder risk and symptomatology in those aged 14 and over so that it can be reliably used by those seeking information for a suspected eating disorder. The development of the tool was predicated on the understanding people now pursue support and information for healthcare concerns on the internet before presenting anywhere else, and the opportunity this presents to inform and encourage help-seeking behaviour in a population largely going undetected in primary healthcare [18]. Although primarily intended for personal use and potential self-referral, the screener is further designed to accurately identify clinical status and thus has the potential to be used in a clinical setting to distinguish true cases from non-cases, with treatment elucidated on that basis.

Questionnaires used in the field of eating disorders have mostly not been validated for online use. Instead, scores on each measure and respective community/clinical norms are derived from paper administration. This is often performed in conjunction with a trained clinician, which as stated, can confound results due to the nature of these types of questions being prone to denial and distortion on face-to-face assessment. Thus, lack of available norms for online administration may impact the findings. However, inclusion of the current gold-standard screening tools against which to validate was determined to be of necessary importance. Additionally, as all surveys are being delivered online in this instance, any effect due to test condition will be homogenous across the survey sample.

This study aims to recruit a large pool of applicants of diverse ethnicity, age, clinical presentation and gender. This is achieved by the use of two recruitment arms – one of which, MTurk, has a broad global reach – and will greatly increase generalisability. It will be one of the first to assess and identify risk on a spectrum rather than classifying true cases and non-cases only, with the aim of improving early intervention in subsyndromal presentations.

The study’s significance will lie in its contribution to e-health early intervention and general screening efficacy across a spectrum of eating disorders. Given the prognostic implications of early intervention and current circumstances surrounding COVID-19, the importance of building e-health resources for an adaptable health system intended to encourage help-seeking cannot be underestimated.

## Data Availability

Not applicable.

## Abbreviations

AN: Anorexia Nervosa
ARFID: Avoidant Restrictive Food Intake Disorder
BED: Binge Eating Disorder
BN: Bulimia Nervosa
EAT: Eating Attitudes Test
ED: Eating Disorder
EDE-Q: Eating Disorder Examination Questionnaire
EDE-QS: Eating Disorder Examination Questionnaire Short
EDI: Eating Disorders Inventory
IOI-S: InsideOut Institute Screener
OSFED: Other Specified Feeding or Eating Disorder
SCOFF: Sick, Control, One Stone, Fat Food questionnaire
MC-SDS: Marlowe-Crowne Social Desirability Scale
CSD-S: Children’s Social Desirability Short Scale
